# A cellular defense memory imprinted by early life toxic stress

**DOI:** 10.1038/s41598-019-55198-4

**Published:** 2019-12-12

**Authors:** Eszter Gecse, Beatrix Gilányi, Márton Csaba, Gábor Hajdú, Csaba Sőti

**Affiliations:** Department of Medical Chemistry, Semmelweis University, Budapest, Hungary

**Keywords:** Cellular neuroscience, Feeding behaviour, Olfactory system, Stress and resilience

## Abstract

Stress exposure early in life is implicated in various behavioural and somatic diseases. Experiences during the critical perinatal period form permanent, imprinted memories promoting adult survival. Although imprinting is widely recognized to dictate behaviour, whether it actuates specific transcriptional responses at the cellular level is unknown. Here we report that in response to early life stresses, *Caenorhabditis elegans* nematodes form an imprinted cellular defense memory. We show that exposing newly-born worms to toxic antimycin A and paraquat, respectively, stimulates the expression of toxin-specific cytoprotective reporters. Toxin exposure also induces avoidance of the toxin-containing bacterial lawn. In contrast, adult worms do not exhibit aversive behaviour towards stress-associated bacterial sensory cues. However, the mere re-encounter with the same cues reactivates the previously induced cytoprotective reporters. Learned adult defenses require memory formation during the L1 larval stage and do not appear to confer increased protection against the toxin. Thus, exposure of *C. elegans* to toxic stresses in the critical period elicits adaptive behavioural and cytoprotective responses, which do not form imprinted aversive behaviour, but imprint a cytoprotective memory. Our findings identify a novel form of imprinting and suggest that imprinted molecular defenses might underlie various pathophysiological alterations related to early life stress.

## Introduction

Associative learning ensures rapid, efficient adaptation to already experienced, re-emerging conditions^1,2^. Re-encountering sensory cues associated with a relevant past experience retrieves the memory and elicits a complex response corresponding to the past incident. Associative memories are generally transient. In contrast, a peculiar learning process takes place early in life during a specific time window, called the critical or sensitive period and gives rise to especially persistent memories. Hence the name, imprinting, which was coined by Konrad Lorenz who observed that newly hatched birds created a strong bond with the first moving object seen^3^. Besides visual cues, olfactory memories driving adult behaviours have been recognized in several vertebrate species, including the homing of salmons to reproduce in the creek they were born^4^ and preference for odours associated perinatally with food in mammals^5,6^. Hence, imprinting serves as the biological basis of secure, long lasting attachment to qualities essential for individual and/or species’ survival. In further support of the profound, life-long impact of imprinted memories, a growing body of evidence shows that facing adversity in the critical period is connected to different cognitive and affective disorders and are accompanied by brain epigenetic, structural and endocrine alterations^7–9^. On the other hand, early life stress predispose to increased telomere erosion, metabolic and cardiovascular diseases in adulthood ultimately affecting healthy lifespan^10–12^. Some of the open questions emerging from these phenomena are whether imprinted memories are involved in somatic conditions shaped by early life adversity, as well as the nature of somatic molecular responses evoked by the retrieval of such memories.

Imprinting is as ancient as learning itself, inherently connected to the appearance of the nervous system. The 959-cell soil-dwelling nematode, *Caenorhabditis elegans* possesses an entirely mapped, invariant network of 302 neurons including a highly sensitive chemosensorium^13^. Yet, this simple nervous system exhibits various forms of learning enabling a surprising behavioural plasticity based on prior experience^14^. For instance, exposure of adult nematodes to an odour in conjunction with food increases preference for the odour. These positive associative memories are spanning from minutes (short term) to over a day (long term) in a nematode life span scale of 2–3 weeks^15^. However, a similar conditioning of newly hatched worms during the L1 larval stage, the critical period, forms an imprinted olfactory memory which is retrievable in 5-day adults^16^. While our work was in progress, recent studies reported that early life exposure to *Pseudomonas aeruginosa* PA14 infection or to the ascr#3 pheromone, respectively, gave rise to imprinted aversive behaviour in adult worms^17,18^. The above findings suggest that early experiences form a long-lasting neural representation and help the animal to avoid imminent threats of pathogenic attacks or population overcrowding.

Pathogens and nutrient scarcity are just a few of adversities that besides behavioural avoidance also require active defense strategies, especially when organismal integrity is injured. In response to various stresses, such as heat, drought, oxidants or toxins, metazoans induce a highly conserved array of specific cellular stress responses. Compared to the immediate behaviour, these cytoprotective transcriptional responses operate on a longer timescale of hours. They restore homeostasis *via* reparation of damage and elimination of the cause, confer increased stress tolerance, boost immunity and promote longevity^19,20^. Moreover, experimental evidence shows a connection between stress responses and neuronal circuitries in *C. elegans*. For instance, the AFD thermosensory neuron not only regulates thermotaxis, but also primes the heat shock transcription factor HSF-1 which enhances survival during heat shock by upregulating the expression of cytoprotective molecular chaperones^21^. Oppositely, disruption of vital cellular processes in somatic cells both stimulates cytoprotective responses and elicits an associative aversive behaviour which requires the cellular stress activated JNK-like kinase pathway^22^.

The findings that neuronal signals facilitate cytoprotective responses, and cellular stress signals underlie learned behaviour indicate a communication and a mutual regulation of somatic and neuronal responses to anticipated danger. Together with the demonstration of imprinted aversion for pathogen and pheromone exposure they raise several questions. Does the exposure to adversities early in life trigger aversive behaviour and cytoprotective responses, respectively? Is imprinted aversion a common consequence of stresses experienced in the critical period? Are cytoprotective responses imprinted and mobilized by associated sensory cues in adults? To address these questions we employed a nematode model of early life stress by exposing worms to toxic compounds, such as antimycin A and paraquat during the L1 stage^22^ and investigated behavioural and cytoprotective responses during development and in adults. We report that early life toxic stresses form a transient aversive memory which is not maintained in adulthood. In contrast, they induce cytoprotective molecular responses that are activated by the re-encounter of adult worms with toxin-associated chemosensory cues. Our study suggests that imprinted cellular defense memories might be involved in the physiological alterations in response to early life stress.

## Results

### Early life toxic stresses induce food aversion behaviour

**Figure 1:**
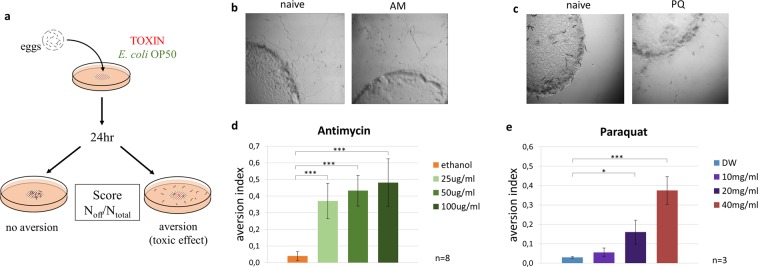
Early life toxic stresses trigger food avoidance behavior. (**a**) Schematic of early life toxin exposure and food aversion assay. Representative images of the effect of antimycin A (AM) (**b**) or paraquat (PQ) (**c**) exposure on food aversion phenomena after 24 hr. Quantification of food leaving behaviours in response to AM (**d**) or PQ (**e**). n = number of independent assays. p values were generated by one-way ANOVA followed by Tukey’s HSD post-hoc correction. *p < 0.05, **p < 0.01, ***p < 0.001.

Adult *C. elegans* exhibits an aversive behavioural response to pathogen-derived sensory cues after an earlier infection that occurred either in adulthood or in the L1 larval stage^17,23^. Likewise, both adults and L3-L4 larvae cease feeding and leave the toxin-contaminated bacterial lawn^22^. We tested whether L1 larvae are able to mount avoidant behaviour in response to toxic stresses by exposing them to a combination of *E. coli* OP50 food source overlaid by antimycin A (AM) or paraquat (PQ). AM is a bacterial toxin, an inhibitor of complex III of the mitochondrial electron transport chain, while PQ is a synthetic herbicide, a reactive oxygen species (ROS) generator^24,25^. Although their chemical structures and mechanisms of action are different, both toxins cause severe damage and compromise mitochondrial energy production. Worms were hatched on *E. coli* OP50 supplemented with the respective toxins and their food leaving behaviour was monitored after 24 hours of toxin exposure (Fig. 1a). We observed that naive L1 larvae remained on the lawn, whereas exposure to either AM or PQ, respectively, induced robust food leaving behaviour (Fig. 1b,c). Thus, L1 larvae are already able to sense toxicity, make a behavioural decision and avoid the otherwise nutritious bacterial lawn. Moreover, dose response curves (Fig. 1d,e) demonstrate that the decision making is proportional to the extent of toxic stress, indicating an ability to carry out an adaptive behavioural response.

### Early life toxic stresses stimulate specific cytoprotective responses

**Figure 2:**
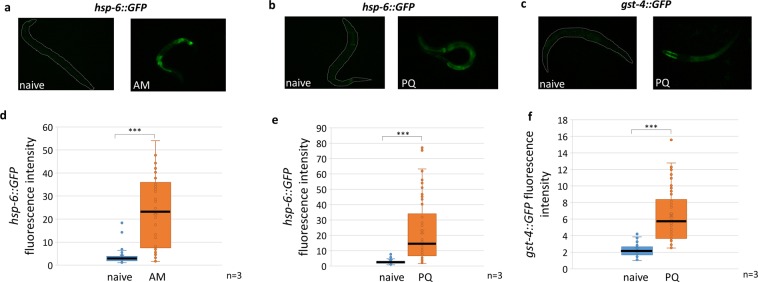
Toxin exposure at the first larval stage induce specific stress and detoxification responses. Epifluorescent microscopic images (**a–c**) representing, and quantification of (**d–f**), the effect of AM exposure on *hsp-6::GFP* (**a**,**d**) and PQ exposure on *hsp-6::GFP* (**b**,**e**) and *gst-4::GFP* (**c**,**f**) reporter expression. GFP expression was imaged and quantified immediately after training. Boxes represent median and first and third quartiles and whiskers represent tenth to 90th percentiles. n = number of independent assays. p values were generated by the non-parametric Kolmogorov-Smirnov test. *p < 0.05, **p < 0.01, ***p < 0.001.

Exposure to toxic stresses trigger a highly conserved array of cytoprotective molecular stress responses that promote organismal survival^26^. Therefore, we employed several GFP reporters to monitor the activation of stress-responsive transcriptional programs. Mitochondrial dysfunction leads to the induction of the mitochondrial unfolded protein response including, *hsp-6*, the mitochondrial paralog of nematode *hsp-70*^27^. Indeed, both AM and PQ exposure enhanced the fluorescence of *hsp-6::GFP* transgenic L1 larvae (Fig. 2a,d and b,e). Stresses might also upregulate drug detoxification responses that neutralize and help excrete toxic chemicals. Assaying two enzymes of glutathione metabolism we found that PQ, but not AM, induced the phase II glutathione S-transferase *gst-4::GFP* (Fig. 2c,f and Fig. S1a), whereas none of them affected the expression of the glutamate cysteine ligase *gcs-1::GFP* (Fig. S1b). Hence, early life stress induced by AM and PQ respectively, gives rise to specific and overlapping cytoprotective responses in L1 larvae (Fig. S1c).

### Lack of early life stress associated aversive memory in adulthood

**Figure 3:**
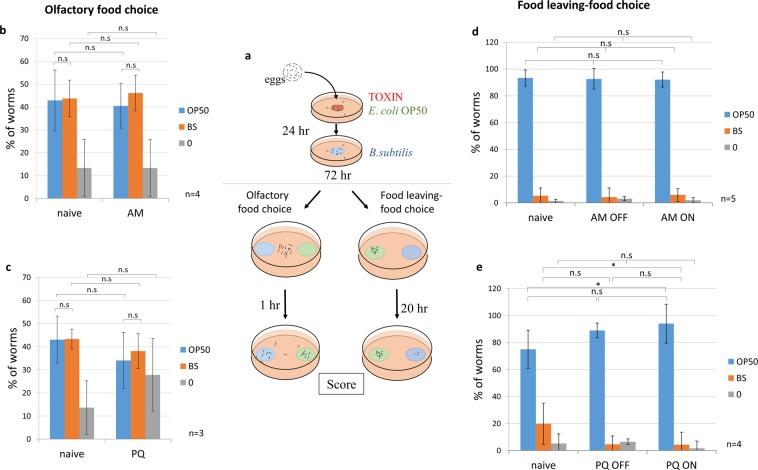
Adult worms do not avoid food sensory cues experienced during early life stress. (**a**) Schematic of the imprinting protocol and the adult olfactory food choice and food leaving-food choice assays. Quantification of adult olfactory food choice after early life exposure to AM (**b**) or PQ (**c**), respectively. Quantification of adult food leaving-food choice after early life exposure to AM (**d**) or PQ (**e**), respectively, in toxin-avoidant (OFF) or non-avoidant (ON) groups of worms. Worms were placed in the middle of the plate in the olfactory and on the *E.coli* OP50 lawn in the food leaving-food choice assay. Choice was quantified by scoring worms on *E. coli* OP50, *Bacillus subtilis* (BS) or on the empty agar surface (0). n = number of independent assays. p values were generated by two-way ANOVA followed by Fisher’s LSD post-hoc correction. n.s, not significant, *p < 0.05, **p < 0.01, ***p < 0.001.

Next, we asked whether the bacterial sensory cues experienced during the toxic insult early in life could evoke an avoidant behaviour in adults. To this end, naive and toxin exposed L1 larvae were washed after 24 hours, transferred to the non-pathogenic *Bacillus subtilis* NRS 231 (BS) strain and grown till adulthood. We employed BS for several reasons. Although *C. elegans* has a very delicate chemosensory system, the sensory cues of the Gram positive BS strain are largely different from those of OP50 allowing a clear distinction between them. Moreover, BS has similar nutritive value, as worms raised on BS appear as healthy as those raised on OP50. Finally, BS is equally attractive compared to OP50 (see choice of naive worms on Fig. 3b,c). We tested adult worms in a classic food choice assay, placing them in the middle of a plate containing OP50 and BS spots at the opposite sides and allowing them to explore for an hour (Fig. 3a left assay). We found that both AM and PQ exposed nematodes exhibited an equal choice between OP50 and BS (Fig. 3b,c). To ensure that these results were not due to any specificity involving the *Bacillus subtilis* strain, toxin exposed worms were grown to adulthood in non-pathogenic *Pseudomonas fluorescens* NCTC 10038 (PF) bacteria. Similarly, we observed no difference between the food preference of AM and PQ treated, compared to naive worms (Fig. S2a,b).

In the above experimental conditions, the choice were based on predominantly olfactory cues. We also used a mixed population of worms that showed toxin-induced aversion or remained on the lawn. We hypothesized that worms that previously avoided the lawn as larvae might have a higher tendency to avoid it as adults, hence avoidant and non-avoidant larvae were separately grown into adulthood. Also, we aimed to better mimic the original sensory experience and to eliminate the negative impact of hunger on the choice in case animals might leave the OP50 lawn. Therefore, we developed a novel, food leaving-food choice assay. Here, using plates containing OP50 and BS bacteria at the opposite ends, worms were placed onto the OP50 lawn and allowed to explore for 20 hours landing on the preferred lawn (Fig. 3a right assay). In this assay, naive worms displayed a much greater preference for OP50 compared to BS probably because they were more likely to remain on the food source available (Fig. 3d,e). Importantly, neither worms that avoided (OFF), nor those that remained on (ON) the toxic lawn during the critical period, altered their preference as adults towards OP50 sensory cues compared to each other or naive nematodes (Fig. 3d,e). Thus, early life stresses induced by AM and PQ do not appear to imprint an associative aversive behaviour.

### Early life stress associated sensory cues revive an imprinted, stress-specific molecular defense memory in adulthood

**Figure 4:**
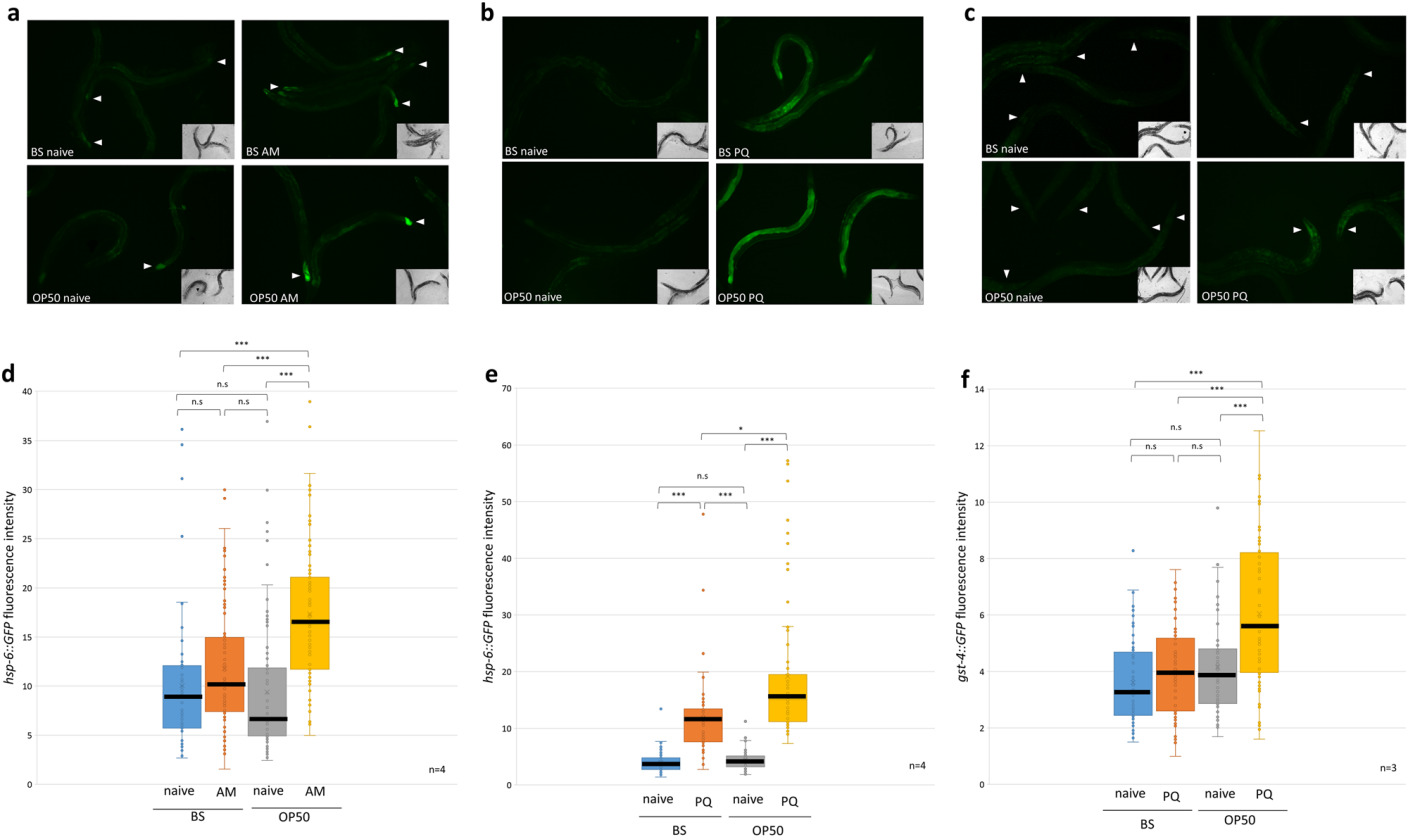
Reactivation of cytoprotective responses in adult worms by early life stress associated sensory cues. Epifluorescent microscopic images (**a**,**b**,**c**) representing, and quantification of (**d**,**e**,**f**), the effect of re-encountering *E. coli* OP50 on the expression of *hsp-6::GFP* in AM treated (**a**,**d**), as well as *hsp-6::GFP* (**b**,**e**) and *gst-4::GFP* (**c**,**f**), in PQ treated adult nematodes, respectively. Boxes represent median and first and third quartiles and whiskers represent tenth to 90th percentiles. n = number of independent assays. Statistics: p values were obtained by the non-parametric Kruskal-Wallis test. n.s, not significant, *p < 0.05, **p < 0.01, ***p < 0.001.

Next, we asked whether a re-exposure of adult worms to the sensory cues co-occurring with the respective early life stresses would induce the corresponding cytoprotective responses. Therefore, after mock and toxin treatment OP50 during the L1 larval stage, GFP reporter strains were grown to adulthood on BS and either placed onto BS or onto OP50 lawn. Reporter expression was examined 24 hours later. *hsp-6*::*GFP* fluorescence was comparable in naive and AM treated worms on BS lawn as well as in naive worms on OP50 showing that reporter expression of adults was neither affected by a previous AM exposure nor by the re-exposure to OP50 (Fig. 4a,d). Strikingly, the re-encounter with OP50 of adult worms exposed to AM or PQ in the L1 stage induced a marked *hsp-6::GFP* expression predominantly in the tail region (Fig. 4a,d). Although *hsp-6::GFP* fluorescence remained higher in PQ treated worms, OP50 sensory cues significantly stimulated it throughout the whole body (Fig. 4b,e). Likewise, a combination of early life PQ exposure and a re-encounter with OP50 significantly increased *gst-4*::*GFP* expression, especially in the proximal head-pharynx region (Fig. 4c,f). Toxin treatments in the L2 developmental stage do not result in elevated *hsp-6::GFP* and *gst-4*::*GFP* expressions in adult worms by the OP50 (Fig. S3a–d). Our findings demonstrate that sensory cues experienced during toxin exposure during, but not after, the critical period re-engage stress-specific cytoprotective molecular responses in adult nematodes. Thus, early life stress gives rise to an imprinted cellular defense memory that is preserved in adulthood.

### Early life stress induced adult stress tolerance is not further enhanced by imprinted memory retrieval

**Figure 5:**
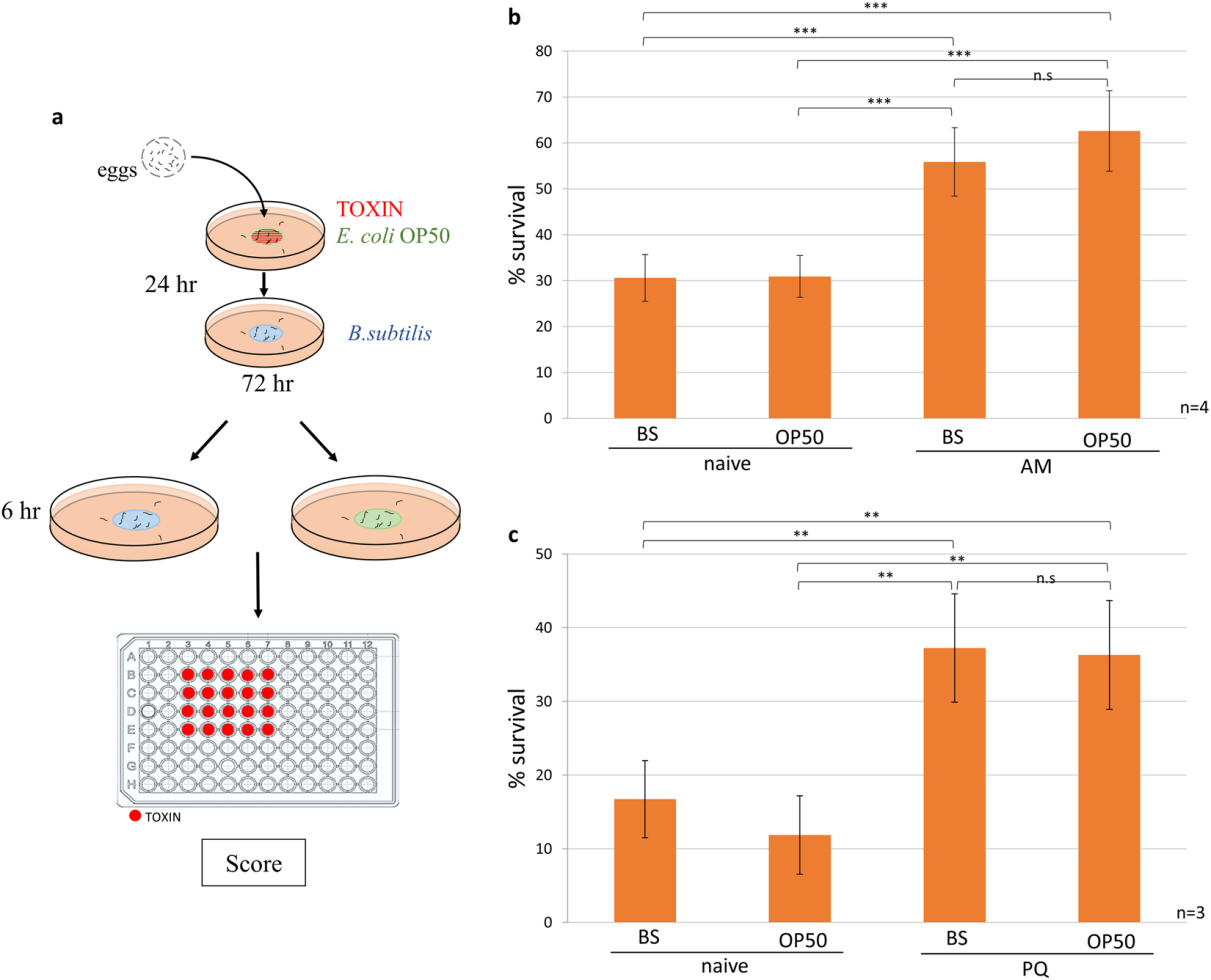
Early life stress induced adult stress tolerance is not further enhanced by imprinted memory retrieval. (**a**) Schematic of the adult toxin tolerance assay. Effect of early life AM **(b)** or PQ **(c)** exposure and the re-encounter with toxin-associated OP50 cues on the survival rates of adult worms subjected to the same toxins. n = number of independent assays. p values were generated by ANOVA followed by Fisher’s HSD post-hoc test. n.s, not significant, *p < 0.05, **p < 0.01, ***p < 0.001.

Finally, we investigated how early life toxin exposure *per se* and reactivation of the imprinted defense memory interferes with the toxic stress tolerance of adult worms. To address these questions, adult worms exposed to AM or PQ on OP50 bacteria in the L1 stage were placed onto plates containing BS or OP50 lawn six hours prior to a lethal toxic stress using the same toxins (Fig. 5a). Toxin exposure during the L1 stage induced an approximately twofold increase in survival compared to naive animals (Fig. 5b,c). However, the re-encounter with OP50 neither altered survival rates in naive nor in toxin-imprinted worms (Fig. 5b,c). Thus, early life toxin exposure at the doses employed induces a lasting and robust stress tolerance in adulthood, which is not further enhanced by retrieval of the imprinted memory.

## Discussion

**Figure 6:**
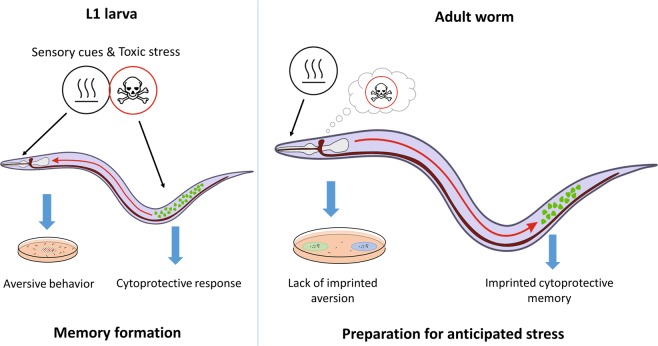
Model of the imprinted cellular defense memory. Exposure of L1 larvae to toxic stresses during the critical period results in tissue damage and toxin-specific systemic cytoprotective responses (green dots). Somatic cells transmit the stress-responsive signals to the nervous system through unknown pathways (red arrow pointing from body to head) which are integrated with the experience of toxicity and the co-occurring sensory cues, giving rise to toxin-induced aversive behaviour and an associative memory formation. Re-encounter of the adult worm with the previously experienced sensory cues retrieves the stored stressful memory, which alerts the somatic cells through unknown pathways (red arrow pointing from head to body). Memory retrieval does not evoke aversion, but reactivates the previously induced cytoprotective responses (green dots). The induction of imprinted cellular defenses might be an attempt to help the worm prepare for impending threats.

In this study, we have established a novel experimental paradigm of early life stress and imprinting by exposing newly hatched *C. elegans* to toxic chemicals. We have shown that early life exposure to toxic stresses induces both locomotory avoidance of the toxin-containing food as well as toxin-specific systemic cytoprotective responses in L1 larvae. This experience, however, does not form a persistent aversive behavioural memory, but imprints a cytoprotective memory that is reactivated by re-encounter of adults with toxin-associated bacterial sensory cues (Fig. 6).

*C. elegans* is a precocial animal living in the soil, where nutriments may be contaminated by dangerous chemicals, including microorganismal, and recently, industrial toxins. Previous studies using toxic chemical exposure during development have shown specific stress responses^28,29^ or both behavioural alterations and stress responses^22,30^ in the L4 or adult stages, respectively. Our results show that worms exposed to toxic doses of either antimycin A (AM) or paraquat (PQ) in the L1 stage are already able to mount protective responses both at the individual intracellular as well as the organismal behavioural level (Figs. 1 and 2). These findings indicate a combined defense strategy including passive behavioural and active molecular coping elements during early development and are consistent with the robust adaptation and evolutionary success of *C. elegans*. Likewise, both locomotory and cellular responses are already mature in the critical period, thus available for neuronal integration to form long-lasting memories.

Indeed, behavioural aversion can be imprinted in *C. elegans*. L1 larvae undergoing a *Pseudomonas aeruginosa* pathogen attack^17^ or exposed to the ascr#3 pheromone^18^ develop an (enhanced) avoidance to these cues as adults, suggesting that adversities in the sensitive period could give rise to a permanent aversive memory. In our experiments, despite a potent aversive behaviour of L1 larvae, adult nematodes did not exhibit avoidance of the OP50 sensory cues that co-occurred with the toxic stress (Figs. 1 and 3). Our experiments yielded identical results, using two unrelated toxins and two unrelated bacterial strains in the classical olfactory food choice assay (Fig. 3a and S2). Further, even after separation of the avoidant and non-avoidant populations and employing a novel assay mimicking the original conditions and eliminating the negative impact of hunger on the choice, nematodes did not show aversive behaviour (Fig. 3d,e). Toxin-exposed worms after recovery were feeding normally, developed into healthy adults, and showed no increased choice of the unpopulated places of the choice plates, therefore, a uniform imprinted aversion towards shared components of bacterial food is also highly unlikely. Albeit the reason is unclear, it appears that imprinted aversion is not a common consequence of aversive insults in *C. elegans*. Nevertheless, imprinting of other stress-induced behavioural alterations might be plausible, which require further studies.

Contrary to the lack of aversion, adult worms with an early life history of toxin exposure activate stress and detoxification genes induced by the respective toxins and only upon meeting the sensory cues they experienced during stress (Fig. 4). Both AM and PQ are potent toxins and induce severe damage^24,25^, hence an organismal dysregulated state might cause random activation of inducible genes. However, the down-regulation of toxin-specific cytoprotective responses, as well as the normal health status and behaviour of these worms argues against toxin-induced metabolic or homeostatic alterations. The reactivation of the same responses in a sensory cue-dependent manner in the absence of toxic stress indicates a regulated neuronal process eliciting a systemic defensive response based on prior experience. Although the signaling pathways and neuronal circuits demand further systematic studies to be identified, our findings indicate an associative learning mechanism and are consistent with two recent studies which showed the systemic activation of intracellular HSF-1- and DAF-16-dependent stress responses, respectively, merely by PA14- or starvation-associated olfactory cues^31,32^. Moreover, cellular stress signals through a JNK-like pathway or *hsf-1* are required for aversive behaviour to occur in stressful conditions^22,31^. Altogether these studies expand our horizon and show that beneath the visually salient behaviour, learned responses exist at the molecular level and involve subliminal, but systemic and highly influential changes affecting among others, behaviour.

Previous studies on associative learning and imprinting, except for the two abovementioned studies^31,32^ mainly investigated behavioural outcomes^14^. We observed a learned associative cytoprotective memory which formed exclusively during the L1 stage and was recalled by toxin-associated sensory cues in adults (Figs. S3, 4). The involvement of the critical period, the specific sensory component and the enduring response identifies a hitherto unknown form of sensory imprinting. To distinguish from behavioural phenomena and underline its location, we refer to it as an “imprinted cellular defense memory” the model of which we depicted in Fig. 6. Interestingly, a 24-hour starvation combined with a specific odour also forms a long-term memory inducing DAF-16 translocation that persists beyond 48 hours^32^. However, the same associative memory could not be imprinted during the L1 stage, indicating that early life stresses do not inevitably give rise to imprinted cellular defenses^32^ (and our study) in agreement with their selective impact on aversive behaviours^17,18^ (and our study). It remains to be investigated, whether other stresses, such as pathogenic attack or population crowding give rise to imprinted cellular defenses and what as yet unidentified factors in which circumstances shape the different forms of imprinted memories.

An important question arising from our study is the potential relevance of the cellular imprinting. The induction of stress responses promote survival and confer tolerance to subsequent stresses and even increase longevity^19,20^. Intriguingly, we found a remarkable increase in toxic stress tolerance of adults by early life toxin treatments, indicating this precocial species exhibit a robust and lasting adaptive capacity already early in life (Fig. 5). Stress responses can be co-ordinated^21^ and associated with sensory cues by neuronal circuits to prepare the organism to cope with imminent adversities^31,32^. Imprinting is a deep learning mechanism during early development that ensures adequate life-long behavioural responses instrumental for survival. In this respect the ability to form stable and specific cytoprotective cellular memories besides the attractive and aversive behaviours might be advantageous for optimal fitness throughout life. Contrary to this hypothesis, we have found that re-exposure of adults to stress-associated sensory cues do not further stimulate the otherwise increased resistance against the same stressor in our experimental conditions (Fig. 5). This finding may suggest a futile attempt of neuronal co-ordination that in this case lags behind the highly trained cellular stress responses robustly induced by toxin re-exposure. Alternatively, a fitness advantage conferred by imprinted cellular defense memories might be relevant under different experimental conditions or different stressors, which requires further systematic studies.

The foundations of stress responses and learning are conserved between worms and primates. Therefore, the induction and imprinting of similar cytoprotective responses during the sensitive period is plausible, especially in altricial species, such as humans, where the immature offspring cannot escape due to both physical (immobility) and neuropsychological (suppressed aversive behaviour due to dependence on the caregiver) constraints^7^. Enduring behaviours and somatic changes imprinted by early life exposure to predator or violence appear to play a role in increased resilience in predatory or violent environment of both animals^33^ and humans^34^. However, early life stress is also associated with increased risk of various mental, emotional^8,9,35^ and somatic diseases, including chronic pain syndromes^10–12^ in more secure and stress-free environmental conditions, especially when stresses were associated with independent, otherwise neutral sensory cues. In both the adaptive as well as the maladaptive cases, among other yet unknown effectors, the acute or sustained activation of the hypothalamic–pituitary–adrenal (HPA) axis plays a causative role^36^. It remains to be seen whether imprinted systemic cellular defense memories retrieved by common sensory cues might modulate behaviour and somatic conditions in vertebrates.

## Methods

### Reagents

Antimycin A, paraquat and other reagents were obtained from Sigma-Aldrich. *Bacillus subtilis* subsp. *spizizenii* NRS 231 (ATCC^®^ 6633^™^) and *Pseudomonas fluorescens* NCTC 10038 (ATCC^®^ 13525^™^) bacteria were from the National Center of Epidemiology, Budapest, Hungary.

### *C. elegans* strains, maintanence and reagents

All strains were obtained from Caenorhabditis Genetics Center. Standard methods were used for maintaining *C. elegans* strains^37^. Worms were grown on *Escherichia coli* OP50 bacteria at 20 °C. The following *C. elegans* strains were used in this study: N2 wild type, SJ4100 zcls13 [hsp-6::GFP], MJCU017 kIs17 [gst-4::GFP, pDP#MM016B]X.

### Imprinting training

Worms were synchronised by placing 15–20 hermaphrodites onto 3 cm diameter NGM plates seeded with 50 µl OP50 bacteria in the middle of the plate and allowed to lay eggs for 4 hours. Before synchronizing on each test plates OP50 bacterial lawn were dropped with 20 µl toxin or the appropriate solvent control (ethanol or distilled water, respectively). If not otherwise indicated, antimycin (AM) or paraquat (PQ) was used at the concentration of 50 µg/ml or 40 mg/ml, respectively. Hermaphrodites were removed and plates were incubated at 20 °C for 24 hours during the L1 larval stage. For L2 training worms were hatched on *B. subtilis* and after 24 hours were transferred to OP50 layered with AM or PQ and incubated for 24 hours.

### Toxin induced L1 aversion

After imprinting training plates were scored immediately for toxin aversion by counting animals that had left or remained on the food lawn and expressed as the aversion index (N_off_/N_total_). Worms were then washed and placed on plates seeded with either *Bacillus subtilis* or *Pseudomonas fluorescens* and grown to adulthood. For the food leaving-food choice experiment, worms that remained on, or left the toxin-containing OP50 lawn, respectively, were separately washed and grown to adulthood on *Bacillus subtilis*.

### Olfactory food choice assay

9 cm round NGM assay plates were seeded with 30 µl of each bacterial suspension (OD_600_ = 1) and incubated at room temperature for 1 hour. 80–100 naive and trained 4-day old worms were washed from their growth plates with M9 buffer, rinsed three times, and placed in the middle of the assay plates, containing OP50 and either *Bacillus subtilis* or *Pseudomonas fluorescens* on the opposite sides. Distribution of worms was scored after 1 hour. Using a short assay plate preparation time and the experimental incubation time allowed the assay to be determined by olfactory cues.

### Food leaving-food choice assay

9 cm round NGM assay plates were seeded by 700 µl of each bacterial suspension (OD_600_ = 1) and incubated at room temperature for 2 hours. 60–80 4-day old worms: naive, trained that showed (OFF) or did not show (ON) toxin-induced aversion, were washed from their growth plates with M9 buffer, rinsed three times, and placed onto the OP50 lawn of the assay plates containing OP50 and *Bacillus subtilis* on the opposite sides. Distribution of worms was scored after 20 hours. This relatively long incubation time and the direct placement of worms onto the lawn better mimicked the original sensory experience and allowed them to choose food based on both olfactory and gustatory cues.

### GFP reporter expression by fluorescence microscopy

After treatments at least 30 L1 or adult worms per condition were placed on 2% agarose pad and immobilized with 25 mM NaN_3_ dissolved in M9 buffer. Images were taken by a Nikon Eclipse E400 microscope with Diagnostic Instruments SPOT model 1.5.0. camera using a GFP fluorescent filter. Images were captured at 10x and 20x magnification. GFP expression levels were evaluated by ImageJ software.

### Toxic stress tolerance

80–100 naive and imprinted 4-day old worms were washed from their growth plates with M9 buffer, rinsed three times, and placed on plates seeded by OP50 or *Bacillus subtilis* for 6 hours. Worms were transferred to a 96-well plate containing 174 µg/ml AM or 24 mg/ml PQ dissolved in M9, in a total volume of 50 µl per well. Animals were incubated in AM and PQ solution for 24 or 20 hours, respectively. Survival was analysed by scoring spontaneous movements.

### Statistical analysis

L1 behavioural assays were analysed by one-way ANOVA with Tukey’s HSD post-hoc test. Reporter expressions were analyzed by non-parametric tests. Pairwise comparisons in larvae were made by Kolgomorov-Smirnov test, multiple comparisons in adults were made by Kruskal-Wallis test. Multiple comparisons of adult behaviour and adult stress tolerance were analysed by two-way ANOVA with Fisher’s LSD post-hoc test. All analyses were performed using STATISTICA program. Parameters of the detailed statistical analyses can be found in Supplementary Table S1. Data were expressed as mean ± standard deviation (SD) in behavioural tests and stress tolerance assays showing normal distribution, or median and first and third quartiles (box) and tenth to 90th percentiles (whiskers) in reporter expression experiments, which displayed non-normal distribution. Statistical levels of significance are as follows: *p < 0.05; **p < 0.01; ***p < 0.001.

## Supplementary information

Supplementary Information
