# Supplementary material for: A cellular defense memory imprinted by early life toxic stress
Source: Sci Rep. 2019 Dec 12;9:18935. doi: 10.1038/s41598-019-55198-4 (PMC6908573; doi:10.1038/s41598-019-55198-4)
Supplement: Supplementary file 1 — Supplementary Information [file 41598_2019_55198_MOESM1_ESM.pdf]

A cellular defense memory imprinted by early life toxic stress

Supplementary Information

Eszter Gecse, Beatrix Gilányi, Márton Csaba, Gábor Hajdú & Csaba Sőti*

Department of Medical Chemistry, Semmelweis University, Budapest, Hungary

Content:

Supplementary Figures S1-S3

Detailed statistical description:

Supplementary Table S1

*Corresponding author. Department of Medical Chemistry, Semmelweis University, P.O.Box 2, Budapest, H-1428, Hungary. Tel.: + 36 1 4591500 extn. 60130; Fax: + 36 1 4591500 extn. 60141; E-mail: soti.csaba@med.semmelweis-univ.hu

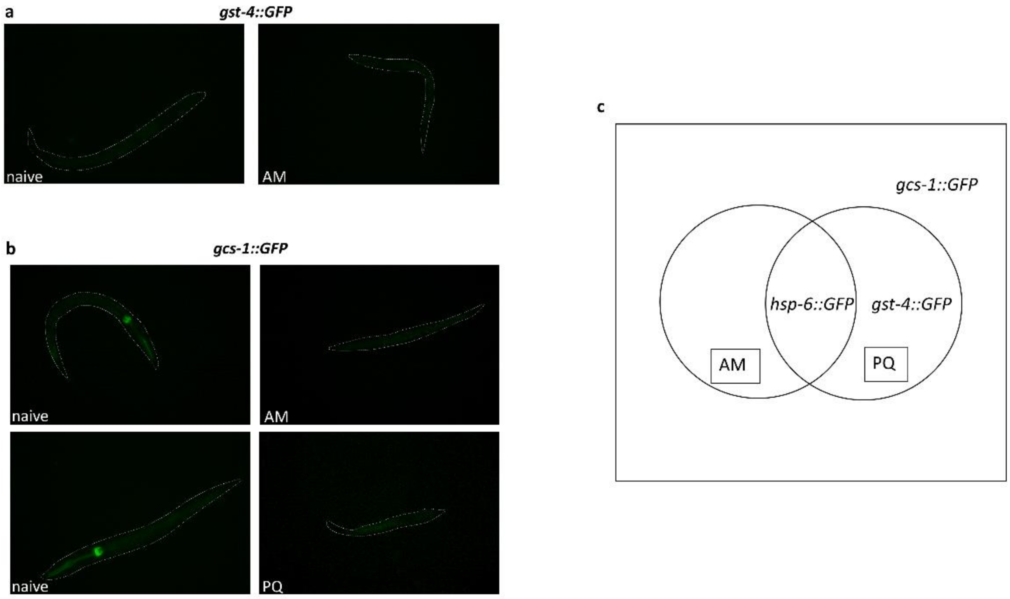

Supplementary Figure S1. Specificity of toxin induced stress and detoxification responses, Related to Figure 2

AM does not induce *gst-4::GFP* (**a**), whereas both AM and PQ appear to slightly inhibit the baseline expression of *gcs-1::GFP* (**b**), reporters in L1 larvae. To confirm the lack of activation, both AM (100 µg/ml) and PQ (40 mg/ml) was employed at the highest concentration that induced aversive behavior. (**c**) Venn diagram showing the AM and PQ induced genes in L1 larvae.

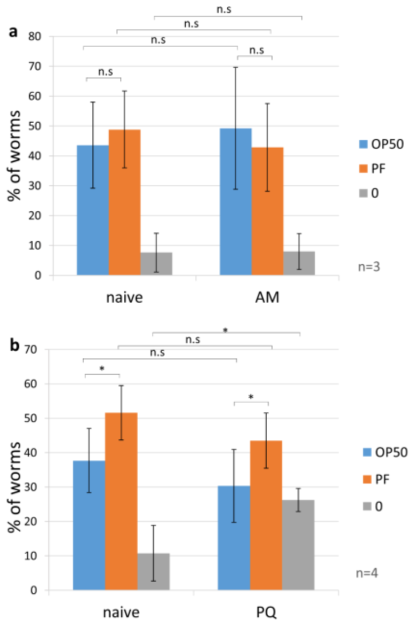

Supplementary Figure S2. Adult worms do not avoid food olfactory cues experienced during early life stress, Related to Figure 3

Early life exposure to AM (**a**) or PQ (**b**), respectively, does not elicit aversive behavior towards OP50 in olfactory food choice assay. Choice was quantified by scoring worms on OP50, *Pseudomonas fluorescens* (PF) or on the empty agar surface (0). n = number of independent assays. n.s, not significant. Data were analysed by two-way ANOVA followed by Fisher's LSD post-hoc correction. n.s, not significant, *p<0.05, **p<0.01, ***p < 0.001.

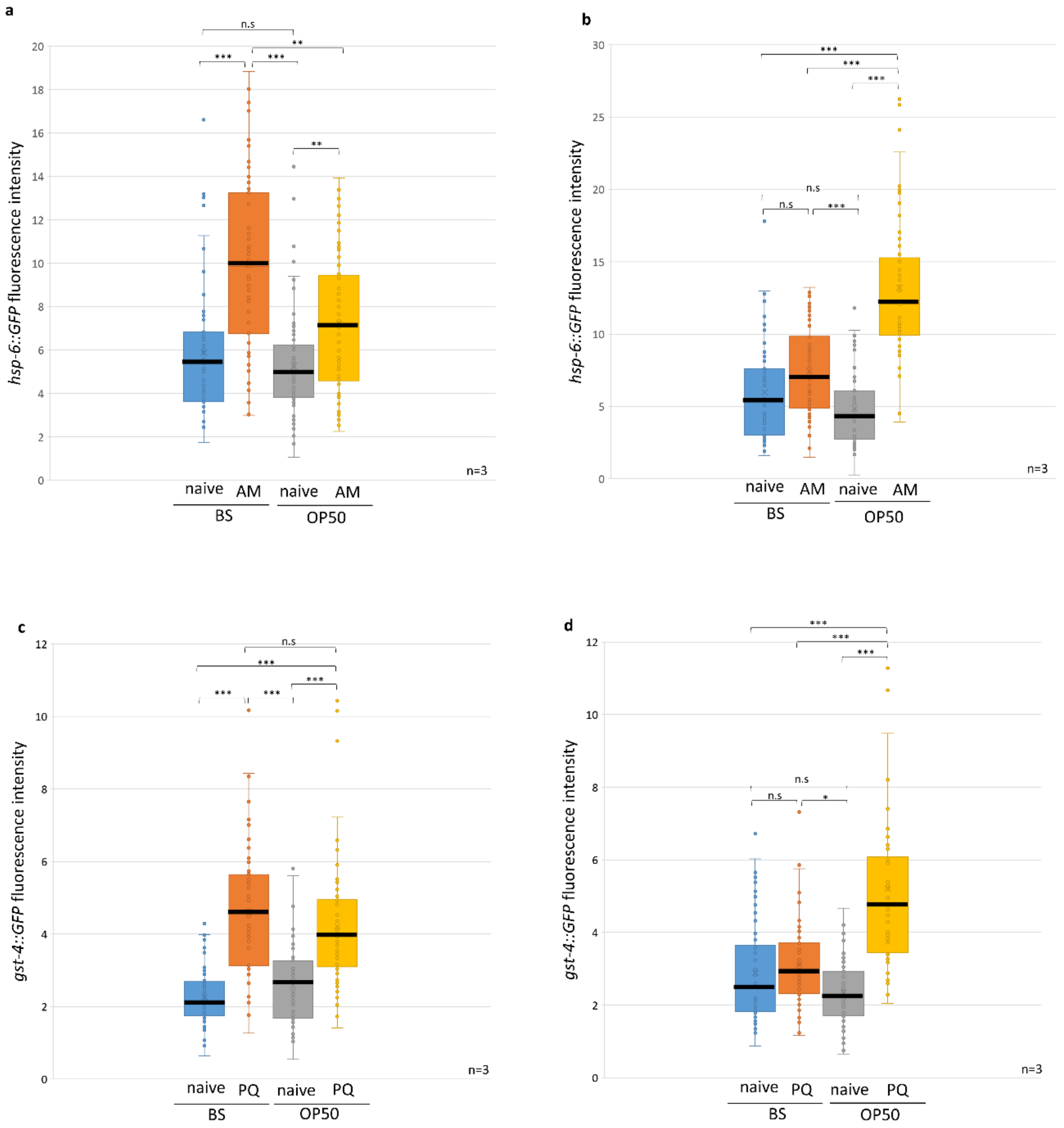

Supplementary Figure S3. Reactivation of cytoprotective memory in adults requires toxin exposure during, but not after, the L1 stage, Related to Figure 4

Quantification of the effect of re-encountering *E. coli* OP50 on the expression of *hsp-6::GFP* or *gst-4::GFP* in adult worms treated with AM or PQ in L2 (**a, c**) or in L1 stage (**b, d**), respectively. Boxes represent median and first and third quartiles and whiskers represent tenth to 90th percentiles. n = number of independent assays. p values were generated by the non-parametric Kruskal-Wallis test. n.s, not significant, *<0.05, **p<0.01, ***p < 0.001.

Detailed statistical description:

Related to **Figure 1.** Data were analysed by one-way ANOVA followed by Tukey's HSD post hoc correction.

Related to **Figure 3.** Data were analysed by two-way ANOVA followed by Fisher's LSD post-hoc correction without interaction analyses because there was no significant interaction between toxin treatment and the distribution of worms. We found significant difference in the distribution of woms between empty agar surface (0) and bacteria (OP50 or BS) but no significant difference between OP50 and BS.

Related to **Figure 5.** Data were analysed by two-way ANOVA followed by Fisher's LSD post-hoc correction without interaction analyses because there was no significant interaction between toxin treatment and re-encountering OP50 or BS bacteria prior to the tolerance assay. We found significant difference according to the early life treatment but no significant difference in type of bacteria re-encountering prior to the assay.

Related to **Supplementary Figure S2.** Data were analysed by two-way ANOVA followed by Fisher's LSD post-hoc correction without interaction analyses because there was no significant interaction between toxin treatment and the distribution of worms. We found significant difference in the distribution of woms between empty agar surface (0) and bacteria (OP50 or BS) but no significant difference between OP50 and BS.

Supplementary Table S1. Parameters of detailed statistical analyses

			degree of freedom	F value	p value
**Figure 1.**	**d**		3	30.627	0.05
	**e**		3	30.251	0.05
**Figure 3.**	**b**	treatment	1	0.001	1
		distribution	2	20.559	0.001
	**c**	treatment	1	0.001	1
		distribution	2	6.159	0.014
	**d**	treatment	2	0.057	0.944
		distribution	2	1594.744	0.001
	**e**	treatment	2	0.001	1
		distribution	2	252.79	0.001
**Figure 5.**	**b**	treatment	1	60.826	0.001
		bacteria	1	0.068	0.797
	**c**	treatment	1	36.863	0.001
		bacteria	1	0.613	0.455
**Supplementary Figure S2.**	**a**	treatment	1	0.001	1
		distribution	2	16.24	0.001
	**b**	treatment	1	0.001	1
		distribution	2	25.23	0.001
